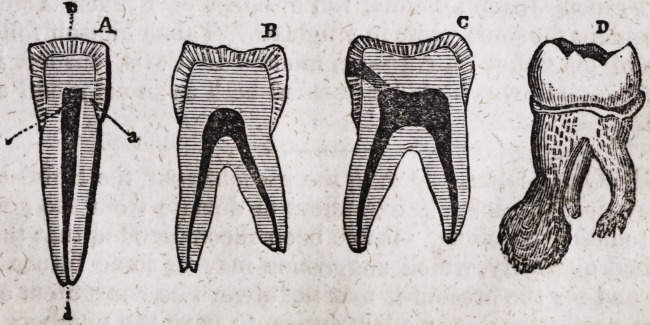# Review

**Published:** 1839-08

**Authors:** Solyman Brown


### REVIEW.

[ BY SOLYMAN BROWN, A. M. ]

"Observations on the Structure, Physiology, Anatomy and Diseases of the Teeth, in two parts; part first by Harvey Burdell, M. D., Honorary Member of the Philadelphia Medical Society, and member of the Medical Society of the City and County of New-York; part second by John Burdell, Dentist. With drawings and Illustrations. New-York: published by Gould & Newman, 1838; pp. 96."

This partnership volume by the brothers Burdell, is a well written and handsomely printed treatise, adapted to the use of the general reader, for whom it was particularly designed. To the well read dentist, even, it is capable of imparting many useful facts, collected from various sources of the first respectability.

The views of Berzelius, Moriani, Bell, Hunter, Cuvier, De Bloinville, Fox, Meckel, Sabatier, Pliny, Lemaire, Camerarius, Bessot, Good, Mayo, Sir Astley Cooper, Lycurgus, Porphyry, Plutarch, Sir Wm. Temple, Cullen, Lord Bacon, Cheyne, Lambe, and Clark, together with other distinguished authors, are happily introduced for the purpose of illustrating the general doctrines of this work.

One fifth part of the volume is taken up with remarks and authorities on the subject of the *natural food of man*, as it stands connected with diseases of the dental organs; and whichever side of this contested question the reader is inclined to espouse, he will be at least amused by this part of the treatise.

But as Mr. John Burdell has illustrated his portion of the volume with several engravings, the use of which he has politely tendered to the publishers of this Journal, we think we cannot do the profession a more acceptable service in this notice, than by introducing the plates together with the explanations which accompany them.

### ANATOMY OF THE HUMAN TEETH.

In the jaw of an adult, perfectly developed, the whole number of teeth is thirty-two, sixteen of which are placed in each jaw. These are divided into four classes—the incisors, the cuspids, the bicuspids, and the molares; and last of all, appear what are commonly called the wisdom teeth, but which are of the last mentioned class.

The incisors, or cutting teeth, numbered in the plate, fig. 1—2, occupy the centre of the jaw in front of the mouth. They are thus termed from a Latin word, which means *to incise*, or cut, because they cut the food. Next come the cuspids, fig. 3; these are the longest of all the teeth, and are commonly called the eye teeth; these, with the bicuspids, fig. 4—5, which stand next to them, form a regular gradation between the incisor and molar teeth. Next are the molares, or grinding teeth, three on each side, above and below, having five prominences with corresponding depressions, perfectly adapted to those opposite them, like mill-stones in miniature, for our convenience in grinding our food.

The following drawing shows the upper and under jaws, with the external alveolar processes cut away.

Drawn by John Burdell, Dentist.

The nerves in the above plate, represented by white or thread-like filaments, which supply the teeth, are important branches of the fifth pair, which has its origin in the brain.

The following drawing (fig. 3,) shows the crystals disposed in radii, springing from the centre of the tooth, so that the extremities of the crystals form the external surface of the tooth, while the internal extremities are in contact with the bony substance, (fig. 2.)

This plate shows a magnified section of a tooth, to illustrate the arrangement of the fibrous crystals composing the enamel. 1. Cavity of the tooth. 2. Bony substance. 3. Enamel, showing the crystals disposed in radii.

Drawn by John Burdell. Dentist.

The above plate represents a side view of the upper and under jaw, with the external alveolar sockets removed. The front, upper and under tooth, which appears darker than the others, belongs to the other side of the jaws. After the superior Maxillary branch passes through the foramen of the skull, it is subdivided, giving off two twigs to different parts of the face, as well as to each root of the upper jaw teeth. The Maxillary nerve of the lower jaw is subdivided, not only at the place where it enters the inside of the jaw near the ear, but at about half its length, where it is seen passing out of the foramen of the external alveolar plate; these twigs are distributed to the lips and integuments of the lower part of the face.

The permanent teeth are arranged in the following manner: 1st. The two central incisors, or the two most prominent teeth in front of the mouth. 2nd. The two lateral incisors, one on each side of the central, which are smaller and not as wide. 3d. The cuspid, or eye teeth, being pointed, one on each side. 4th. The bicuspids, or small double teeth, two on each side. 5th. The molares, or what are called grinding teeth, two on each side, and much larger and stronger than the others. 6th. The *dentes sapientiæ*, or wisdom teeth, one on each side, and smaller than the molares; these teeth do not appear until about adult age, and are not as dense as the others, and often commence decaying soon after they appear. When the whole set is complete there are as many teeth in the under as the upper jaw, and the above arrangement will apply to either.

The following drawing exhibits the jaws, with several of the teeth in a diseased state, as they are situated in the maxillary bones,—the fleshy covering, or soft parts, being removed to show more accurately the parts affected.

Diseased Teeth.

The teeth numbered in the above plate are all affected with caries, which has penetrated to the nerve, with the exception of fig. 3, which is in a natural and healthy condition. Fig. 1, 4, and 7, show the jaw and teeth in an incipient stage of disease, the ulcer, or abscess having just commenced, and not yet progressed as far as the others.

Fig. 6 and 9 are equally affected at the roots; fig. 6 shows the effect of the ulcer on the jaw after a long continuance of the disease. Fig. 9 shows part of the bone or alveoli removed, to expose the ulcer which has formed at the root. Fig. 8 and 2 are similarly affected. The bone is removed from fig. 5, to show the ulcer when first commencing.

The white lines from the roots of these teeth are twigs or branches of the subdivisions of the fifth pair of nerves, as I have heretofore explained.

In the above plate, A shows a central incisor split through the centre of its body; *a* the bony part; *b* the enamel; *c* the internal nerve; *d* the medium of communication between the nerve of the jaw and of the tooth.

B shows a molar or double tooth of the lower jaw, of an individual aged about 40, split through the centre to show the comparative size of the nerve at this age; and that marked C exhibits a tooth of a person at the age of about 20, to show that this nerve is much larger than in an advanced age, as I have stated in another part of this work; it can likewise be seen that this tooth has commenced decaying at the side, but has not so far advanced but that it could be preserved if carefully and properly plugged. D shows a diseased upper tooth, after the alveolar abscess or sac has formed at the extremity of the root, as I have exhibited in the drawing of diseased teeth.

## Figures and Tables

**Figure f1:**
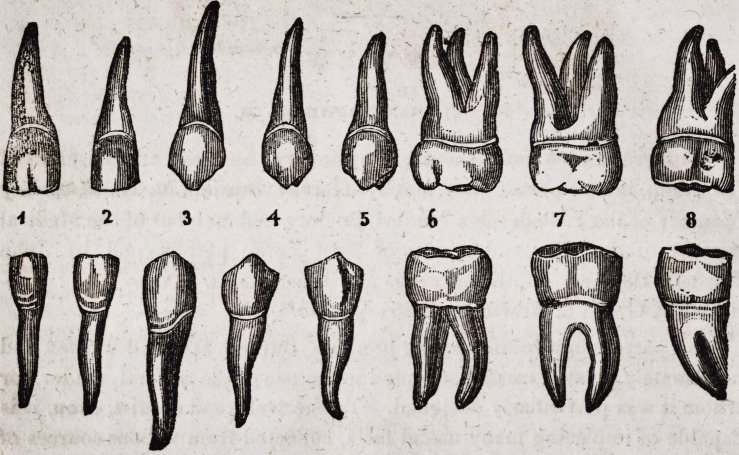


**Figure f2:**
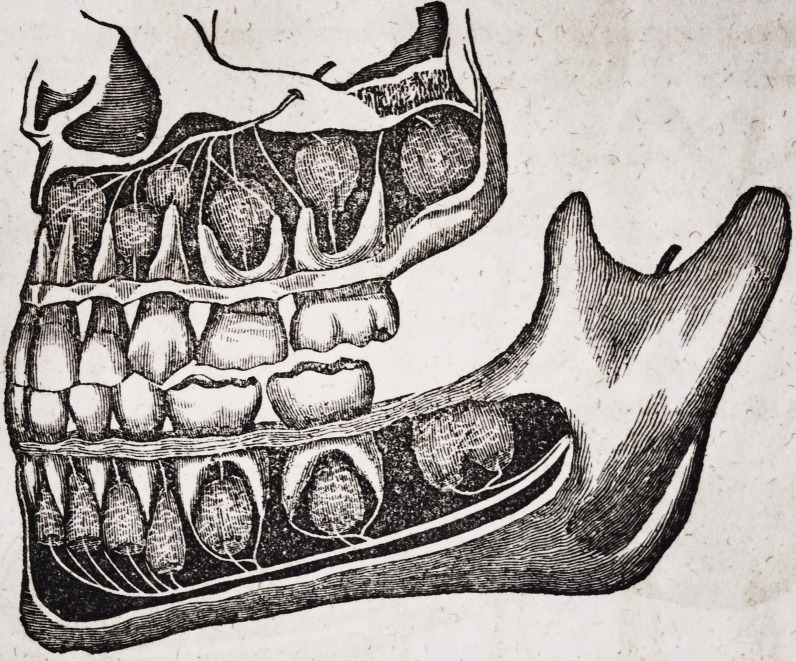


**Figure f3:**
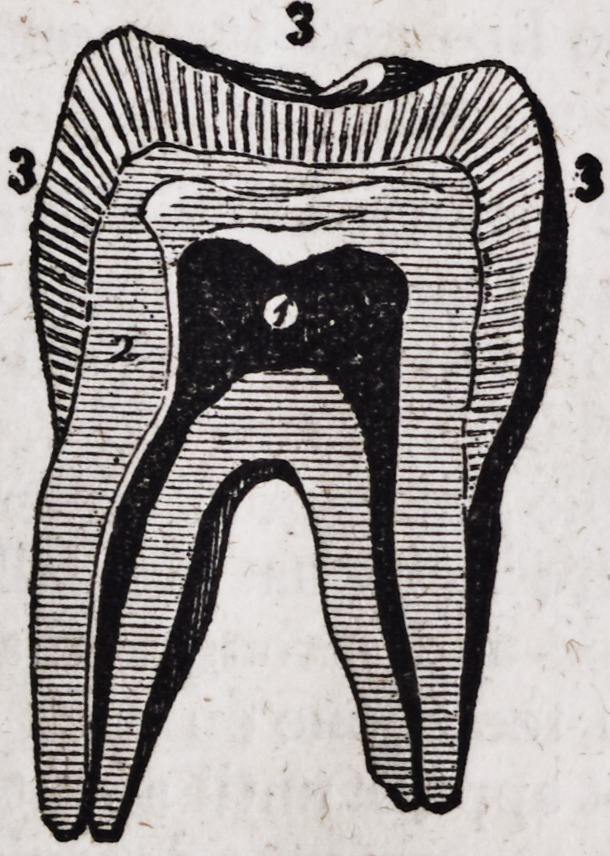


**Figure f4:**
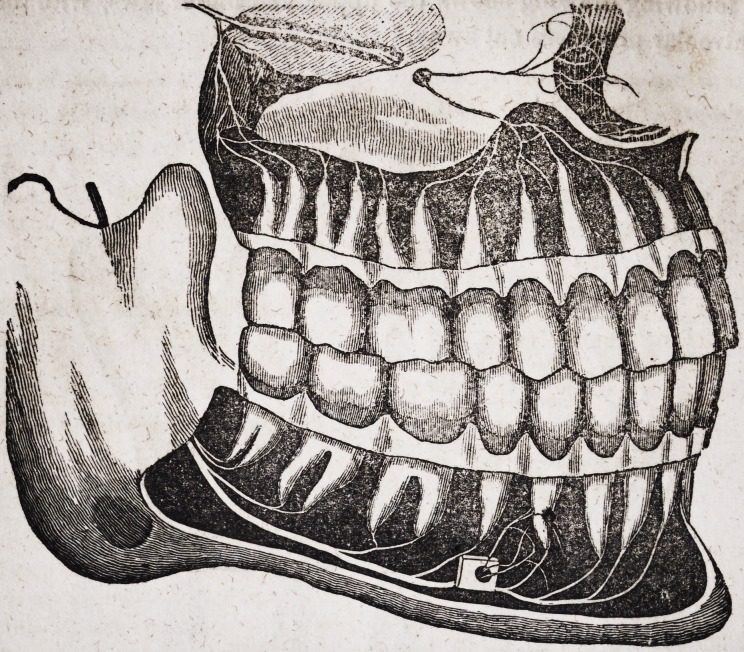


**Figure f5:**
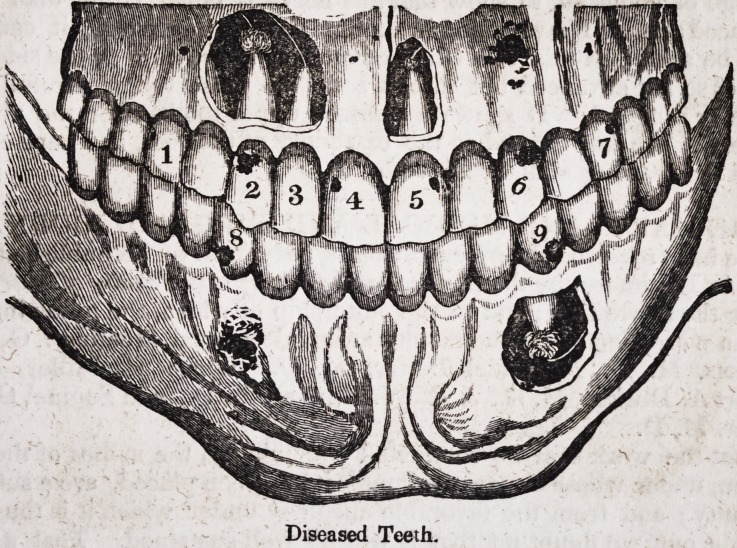


**Figure f6:**